# Reconexão das Veias Pulmonares Pós Ablação. Um Desafio a ser Superado

**DOI:** 10.36660/abc.20210438

**Published:** 2021-07-15

**Authors:** Silvia Helena Cardoso Boghossian, Ricardo Mourilhe-Rocha

**Affiliations:** 1 Universidade do Estado do Rio de Janeiro Rio de JaneiroRJ Brasil Universidade do Estado do Rio de Janeiro, Rio de Janeiro, RJ – Brasil; 2 Americas Medical City-Hospital Vitória Hospital Samaritano-Barra Rio de JaneiroRJ Brasil Americas Medical City-Hospital Vitória e Hospital Samaritano-Barra, Rio de Janeiro, RJ - Brasil; 3 Hospital Pró Cardíaco Rio de JaneiroRJ Brasil Hospital Pró Cardíaco, Rio de Janeiro, RJ - Brasil

**Keywords:** Reconexão, Veias Pulmonares, Ablação, Crioablação, Fibrilação Atrial

A fibrilação atrial (FA) é a arritmia sustentada mais comum na população e sua incidência aumenta significativamente com a idade.^1^ Com o envelhecimento da população estima-se um incremento significativo na prevalência da FA, e seu controle adequado tem se tornado um grande desafio.

Durante décadas, até final dos anos 90, a opção terapêutica disponível e mais utilizada na reversão e prevenção de recorrências da FA eram os fármacos antiarrítmicos (AA) e a cardioversão elétrica; entretanto, o tratamento clínico com fármacos AA, se mostrou pouco eficaz na manutenção do ritmo sinusal, com índice de recidiva acima de 50%.

O entendimento dos mecanismos envolvidos na gênese da FA através da tecnologia de mapeamento atrial, bem como a pouca efetividade dos fármacos AA e a elevada prevalência da FA impulsionaram pesquisas na busca de novas opções terapêuticas, para controle dessa arritmia.

A descoberta de que focos ectópicos situados no interior das veias pulmonares (VP) poderiam deflagrar e perpetuar a FA, inaugurou uma nova era no tratamento dessa arritmia.

Nos últimos anos, em razão dos diversos estudos demonstrando a eficácia e segurança do procedimento de ablação, a indicação do tratamento não farmacológico vem sendo cada vez mais frequente e precoce.

Independentemente do tipo de energia ou da técnica utilizada, o isolamento completo das VP, é reconhecidamente base fundamental para o tratamento não farmacológico da FA e, portanto, tem sido recomendado como passo inicial, na ablação de FA em diretrizes nacionais e internacionais.^1 - 3^ Inicialmente, essa técnica era indicada apenas na FA paroxística, até que estudos subsequentes demonstraram sua não inferioridade em relação a outros procedimentos mais complexos e abrangentes, em pacientes com FA persistente.^4^

Atualmente o isolamento das VP pode ser realizado por meio da energia de radiofrequência com aplicações focais ponto a ponto ou por meio de congelamento, utilizando o balão de crioenergia.

Embora amplamente utilizada na Europa e América do Norte, apenas esse ano a tecnologia de crioablação foi regulamentada pela ANS para utilização em território nacional.

O Estudo “Fire and Ice”, foi o primeiro grande estudo multicêntrico randomizado comparando os resultados do uso do criobalão e da energia de radiofrequência na ablação da FA paroxística e demonstrou que as tecnologias foram semelhantes, tanto em relação a eficácia quanto à segurança. Em uma análise secundária deste estudo, o criobalão apresentou menor taxa de reospitalização e re-intervenção.^5^ Posteriormente outros ensaios compararam as tecnologias e demonstraram resultados semelhantes.

Recentemente, dois estudos utilizando a crioenergia, demonstraram a superioridade da ablação como primeira linha de tratamento para controle de FA, quando comparada ao tratamento farmacológico.^6 , 7^

Recentemente publicamos nos Arquivos Brasileiros de Cardiologia, a primeira experiência de um centro brasileiro, com o isolamento elétrico das veias pulmonares (IEVP) utilizando o balão de crioenergia como abordagem inicial para o tratamento não farmacológico da FA e demonstramos resultados similares aos obtidos em grandes centros internacionais.^8^

Apesar de todo avanço tecnológico, aproximadamente um terço dos pacientes, apresentam recidiva de arritmias atriais após um procedimento inicial bem-sucedido. A reconexão da condução nas VP e a ocorrência de focos fora das veias são os dois fatores principais que justificam a recorrência. Atualmente, os índices de isolamento agudo das VP são bastante elevados, e o grande desafio é manter esse isolamento a longo prazo.

Estudos prévios, avaliando pacientes submetidos a reablação após um procedimento inicial bem-sucedido com radiofrequência, demonstraram que a reconexão das VP é o fator dominante para recorrência, visto que entre os pacientes encaminhados a um segundo procedimento, 80% apresentam reconexão de alguma veia.^9^

No presente estudo, Nolasco et al.,^10^ relatam reconexão de VP em 77,8% dos pacientes encaminhados a um segundo procedimento de ablação após IEVP com criobalão, e observaram sítios de reconexão na região antero-superior da VP superior esquerda, e nas regiões septal e inferior da VP superior direita, e atribuíram os achados a uma maior espessura da parede dos átrios, dificultando o contato adequado do balão de crio. Estes achados diferem do estudo de Godin et al.,^11^ que observaram 64% de reconexão das VP em pacientes com FA paroxística encaminhados a segundo procedimento; e identificaram predomínio das lacunas de conexão na porção inferior das veias inferiores, sendo 80% na VP inferior esquerda e 67% na VP inferior direita, e também diferem dos resultados de Kettering et al.^12^ em que a distribuição dos sítios de reconexão foi semelhante entre as veias.

O conhecimento de locais com maior predisposição para reconexão poderia servir de orientação para o desenvolvimento de tecnologias que auxiliem a vencer este grande desafio, no entanto, os resultados discrepantes com relação aos sítios de reconexão nos diferentes estudos não nos permitem definir uma região mais propensa à reconexão, e nos levam a crer que os locais de maior dificuldade para isolamento das veias se relacionam a variações anatômicas individuais.
