# Supplementary material for: Reconexão das Veias Pulmonares Pós Ablação. Um Desafio a ser Superado
Source: Arq Bras Cardiol. 2021 Jul 15;117(1):106–7. [Article in Portuguese] doi: 10.36660/abc.20210438 (PMC8294737; doi:10.36660/abc.20210438)
Supplement: Translation

# Translation: Reconnection of Pulmonary Veins After Ablation. A Challenge to be Overcome

**Authors:** Silvia Helena Cardoso Boghossian, Ricardo Mourilhe-Rocha

**Affiliations:** 1 Brazil Universidade do Estado do Rio de Janeiro, Rio de Janeiro, RJ – Brazil; 2 Brazil Americas Medical City-Hospital Vitória e Hospital Samaritano-Barra, Rio de Janeiro, RJ - Brazil; 3 Brazil Hospital Pró Cardíaco, Rio de Janeiro, RJ - Brazil

**Keywords:** Reconnection, Pulmonary Veins, Ablation, Cryoablation, Atrial Fibrillation

Atrial fibrillation (AF) is the most common sustained arrhythmia in the population and its incidence significantly increases with age.^1^As life expectancy rises, it is estimated that AF prevalence will suffer a significant increase, and its adequate control will continue to be a major challenge.

Until the end of the 90s, the therapeutic options available and most used in the reversion and prevention of AF recurrences were antiarrhythmic drugs (AA) and electrical cardioversion; however, clinical treatment with AA drugs has been shown ineffective in maintaining sinus rhythm, with a recurrence rate above 50%.

The understanding of the mechanisms involved in the genesis of AF through atrial mapping technology, as well as the low effectiveness of AA drugs and the high prevalence of AF, have stimulated research in the search for new therapeutic options to control this arrhythmia.

The discovery that ectopic foci located inside the pulmonary veins (PV) could trigger and perpetuate AF, ushered a new era in the treatment of this arrhythmia.

Due to the various studies demonstrating the efficacy and safety of the ablation procedure in recent years, non-pharmacological treatment is being indicated earlier and more frequently.

Regardless of the type of energy or the technique used, complete isolation of the PV is recognized as a fundamental basis for the non-pharmacological treatment of AF and, therefore, has been recommended as an initial step in the ablation of AF in national and international guidelines.^1 - 3^Initially, this technique was indicated only in paroxysmal AF, until subsequent studies demonstrated its non-inferiority in relation to other more complex and comprehensive procedures, in patients with persistent AF.^4^

Currently, PV can be isolated using radiofrequency energy with focal point-to-point applications, or through freezing, using the cryoprobe.

Although widely used in Europe and North America, it was only this year that cryoablation technology was regulated by the ANS - the national health agency - for use in Brazil.

The “Fire and Ice” Study was the first large randomized multicenter study comparing the results of the use of cryoballoon and radiofrequency energy in the ablation of paroxysmal AF and it demonstrated that the technologies were similar, both in terms of effectiveness and safety. In a secondary analysis of this study, the cryo balloon showed a lower rate of rehospitalization and re-intervention.^5^Subsequently, other trials compared the technologies and demonstrated similar results.

Recently, two studies using cryoenergy have demonstrated the superiority of ablation as the first line of treatment for the control of AF when compared to pharmacological treatment.^6 , 7^

We recently published in the Brazilian Archives of Cardiology the first experience of a Brazilian center using the cryoballoon for pulmonary vein isolation (PVI) as an initial approach for the non-pharmacological treatment of AF, and we demonstrated results similar to those obtained in large international centers.^8^

Despite all the technological advances, approximately one-third of the patients present recurrence of atrial arrhythmias after a successful initial procedure. The reconnection of the PV and the occurrence of foci outside the veins are the two main factors that justify the recurrence. Currently, the rates of acute PVI are quite high, and the major challenge is to maintain this isolation in the long term.

Previous studies evaluating patients undergoing redo ablation after a successful initial radiofrequency procedure have shown that the reconnection of the PV is the dominant factor for recurrence, since, among patients referred for a second procedure, 80% demonstrated reconnection of some vein.^9^

In the present study, Nolasco et al.^10^reported reconnection of PV in 77.8% of patients referred for a second ablation procedure after PVI with cryoballoon, and observed sites of reconnection in the anterosuperior region of the upper left PV, and in the septal and inferior regions of the right upper PV, and attributed the findings to a greater thickness of the atrial wall, hindering the proper contact of the cryo balloon. These findings differ from the study by Godin et al.,^11^who observed 64% of reconnection of the PV in patients with paroxysmal AF referred for the second procedure; and identified a predominance of connection gaps in the lower portion of the lower veins, 80% in the lower-left PV and 67% in the lower right PV - also differing from the results of Kettering et al.^12^which saw that the distribution of the reconnection sites was similar between the veins.

The knowledge of sites with a greater predisposition for reconnection could serve as a guide for the development of technologies that would help overcome this great challenge, however, the discrepant results in relation to the sites of reconnection in the different studies do not allow us to define regions that are more prone to reconnection and lead us to believe that difficulty in achieving permanent PVI is due to individual anatomical variations.
